# Improving the potency of DNA vaccine against Chicken Anemia Virus (CAV) by fusing VP1 protein of CAV to Marek's Disease Virus (MDV) Type-1 VP22 protein

**DOI:** 10.1186/1743-422X-8-119

**Published:** 2011-03-14

**Authors:** Hassan Moeini, Abdul Rahman Omar, Raha Abdul Rahim, Khatijah Yusoff

**Affiliations:** 1 Department of Microbiology, Faculty of Biotechnology and Biomolecular Sciences, Universiti Putra Malaysia, 43400 Serdang, Selangor, Malaysia; 2 Institute of Bioscience, Universiti Putra Malaysia, 43400 Serdang, Selangor, Malaysia; 3 Department of Molecular Biology, Faculty of Biotechnology and Biomolecular Sciences, Universiti Putra Malaysia, 43400 Serdang, Selangor, Malaysia

## Abstract

**Background:**

Studies have shown that the VP22 gene of Marek's Disease Virus type-1 (MDV-1) has the property of movement between cells from the original cell of expression into the neighboring cells. The ability to facilitate the spreading of the linked proteins was used to improve the potency of the constructed DNA vaccines against chicken anemia virus (CAV).

**Methods:**

The VP1 and VP2 genes of CAV isolate SMSC-1 were amplified and inserted into eukaryotic co-expression vector, pBudCE4.1 to construct pBudVP2-VP1. We also constructed pBudVP2-VP1/VP22 encoding CAV VP2 and the VP22 of MDV-1 linked to the CAV VP1. *In vitro *expression of the genes was confirmed by using RT-PCR, Western blot and indirect immunofluorescence. The vaccines were then tested in 2-week-old SPF chickens which were inoculated with the DNA plasmid constructs by the intramuscular route. After *in vivo *expression studies, immune responses of the immunized chickens were evaluated pre- and post-immunization.

**Results:**

Chickens vaccinated with pBudVP2-VP1/VP22 exhibited a significant increase in antibody titers to CAV and also proliferation induction of splenocytes in comparison to the chickens vaccinated with pBudVP2-VP1. Furthermore, the pBudVP2-VP1/VP22-vaccinated group showed higher level of the Th1 cytokines IL-2 and IFN-γ.

**Conclusions:**

This study showed that MDV-1 VP22 gene is capable of enhancing the potency of DNA vaccine against CAV when fused with the CAV VP1 gene.

## Background

Chicken anemia virus (CAV) is a small non-enveloped virus of genus *Gyrovirus *from *Circoviridae *family, which causes anemia in young susceptible chickens and subclinical infections in older chickens [1-3]. Commercially available vaccines against CAV infection which are based on non-attenuated virulent CAV propagated in chicken embryos [4] or attenuated live vaccine [5] cannot be used in chickens in lay and within 21 days of slaughter. Furthermore, live attenuated vaccine may cause clinical disease if not attenuated sufficiently and sometimes spreading of the modified viruses to young chickens may cause the disease. In a recent development, plasmid DNA-based vaccines have emerged as one of the more promising applications of non-viral gene therapy. One such subunit vaccine against infectious chicken anemia was developed by using recombinant baculovirus as a vector for the expression of the CAV proteins [6]. They found that co-synthesis of VP1 and VP2 is required for the induction of neutralizing antibodies.

A limitation in the use of DNA vaccine is its inability to spread *in vivo*. Thus, a strategy to facilitate the spread of the antigen may significantly enhance the potency of the vaccine. This can be demonstrated by the fusion of the VP22 gene of Marek's disease virus type-1 (MDV-1) to the target gene encoding the antigenic protein. It has been shown that the VP22 protein of Marek's disease virus type-1 (MDV-1) possesses the ability to improve DNA vaccine potency by facilitating intercellular spreading of the linked protein [7]. The MDV-1 VP22 is a phosphorylated protein with a DNA-binding activity located at the N terminus of the protein that displays cell trafficking properties [8]. In fact, VP22 is a tegument protein involved in intercellular transport and movement between cells from the original cell of expression into the neighboring cells [9,10].

We therefore investigated the use of MDV-1 VP22 linked to CAV VP1 gene in a recombinant DNA plasmid, namely pBudVP2-VP1/VP22 which allows the CAV VP1 fused to MDV-1 VP22 to be simultaneously expressed with the CAV VP2.

## Material and methods

### Expression vector and viral genes

The pBudCE4.1 co-expression vector (Invitrogen, USA) was used to construct the DNA vaccines. The vector contains the human cytomegalovirus (CMV) immediate-early promoter and the human elongation factor 1α-subunit (EF-1α) promoter for high-level, constitutive, independent expression of two recombinant proteins. The VP22 gene of MDV-1 strain CVI988/Rispens (Accession No: AY311498) and recombinant pCRVP1-VP2 cloning vector containing VP1 and VP2 genes of CAV isolate SMSC-1 (Accession No. AF285882) were obtained from the Faculty of Veterinary Medicine, University Putra Malaysia (UPM).

### Construction of DNA vaccines

In this study, two DNA plasmids, namely pBudVP2-VP1 and pBudVP2-VP1/VP22 were constructed. The VP1 and VP2 genes of CAV were amplified from the recombinant plasmid PCRVP1-VP2 using specific primers for the CAV VP1 and VP2 genes listed in Table 1. Reverse primers were designed without stop codon to insert the genes upstream of the His-tag sequence. The VP1 gene was inserted into the *Not*I and *Kpn*I cloning sites of pBudCE4.1 plasmid under the control of the EF-1α promoter. The VP2 gene of CAV was then inserted into the *Sal*I and *Bam*HI sites of CMV promoter in the pBudVP1 construct to generate pBudVP2-VP1. To construct the pBudVP2-VP1/VP22, the MDV-1 VP22 DNA fragment was amplified using a set of primers listed in Table 1 and then cloned into the *Kpn*I and *Xho*I cloning sites of pBudVP2-VP1 in the frame with the VP1 gene. After DNA transformation into *Escherichi coli *Top10, the inserted genes were verified by restriction-enzyme analysis and PCR. The identical sequence of the inserts was confirmed by double-stranded sequencing. All the plasmid constructs and the control plasmid were purified using a large-scale Endotoxin-free plasmid purification kit (Endo-free Maxiprep Kit, Qiagen, USA).

**Table 1:** Primers for (A): PCR amplification and RT-PCR analysis of the viral genes; (B): RT-PCR analysis of chicken cytokines

Gene	Accession Number	Sequence (5' to 3')		Expected product size (base pair)
**A:**				
CAV VP1	AF285882	Forward Reverse	CTAACGCGGCCGCACCATGGCAAGACGAGCTCGC CTAGGGGTACCCCAGTACATGGTGCTGTTGG	1335
CAV VP2	AF285882	Forward Reverse	GCTAAGTCGACACCATGCACGGGAACGGC CATGGGGATCCCACTATACGTACCGGGGC	648
MDV-1 VP22	AY311498	Forward Reverse	CATGGGGTACCATGGGGGATTCTGAAAGGC GTACGCTCGAGTCGCTATCACTGCTACGAT	728
**B:**				
IL-4	NM_001007079	Forward Reverse	AGCTCTCAGTGCCGCTGATG TAGCTAGTTGGTGGAAGAAGG	321
IL-6	NM_204628	Forward Reverse	ATGAACTTCACCGAGGGCTGC ACGGTCTTCTCCATAAACGAAG	680
IL-12	AY262752	Forward Reverse	ACACATCTGATGAAGCACTGCC TTGGGATATGTCCAGGTC-ACAG	598
β-actin (positive control)	EU931581	Forward Reverse	ATGTGCAAGGCCGGTTTCGC TCCTCAGGGGCTACTCTCAG	254

### In vitro transcription and translation of the constructs

*In vitro *expression of the recombinant plasmids was carried out in chicken MDCC-MSB1 cell line. The MSB1 cells were transfected with the plasmids using Lipofectamine™2000 reagent (Invitrogen, USA) according to the manufacturer's recommendation with some modifications. After 48 h of transfection, expression of the genes was evaluated by RT-PCR, Western blot and indirect immunofluorescence.

Total RNA was extracted from the transfected MSB1 cells using Trizol and then treated with DNase I to remove DNA contamination. To confirm DNA removal from the samples, they were tested by PCR using specific primers for the inserted genes. The RNA from normal MSB1 cells was used as negative control. RT-PCR reactions were performed in 25 μl volume in a Gradient Thermal Cycler (BioRad, USA). Following a reverse transcription step at 45°C for 45 min, the samples were denatured at 94°C for 3 min and amplification was carried out in 35 cycles 94°C for 40 s, 55°C for 1 min and 68°C for 2 min; and a final elongation step at 68°C for 10 min. RT-PCR products were then analyzed by electrophoresis on a 1% agarose gel.

*In vitro *translation of the genes was studied by Western blotting using chicken anti-CAV serum and mouse anti-His monoclonal antibody (Promega, USA) as primary antibodies. Expression of the genes was also evaluated by immunofluorescence test according to Richter and Wick [11] using chicken anti-CAV serum as primary antibody and fluorescein isothiocyanate (FITC)-conjugated anti-chicken IgY (Promega, USA) as secondary.

### Evaluation of DNA vaccines in SPF chickens

Specific pathogen free (SPF) chicken eggs taken from Malaysian Vaccines & Pharmaceuticals Sdn Bhd, Malaysia were hatched and maintained under specific-pathogen free condition with free access to feed and water. Two-week-old SPF chickens (n = 10) were vaccinated with 150 μg of pBudVP2-VP1, pBudVP2-VP1/VP22, parental plasmid or 1x PBS via intramuscularly injection. Chickens were boosted two times with the same regimen as the first injection, at 2 weeks intervals. Blood was withdrawn from the wing vein of the chickens before and 10 days after the last injection and collected sera were kept in -20°C for further analysis. All procedures were conducted with the protocols approved by Animal Care and Use Committee of the Faculty of Veterinary Medicine, University Putra Malaysia (UPM).

### In vivo expression analysis

Transcriptional expression of VP1, VP2 and MDV-1 VP22 genes was determined in the vaccinated groups by RT-PCR using the specific primers. Ten days after the last vaccination, the chickens were sacrificed and their skeletal muscle (at the site of injection) was harvested; homogenized and their total RNA was extracted. After treatment with DNase I, DNA removal was confirmed by PCR and then DNA-free samples were subjected to RT-PCR as described above.

Western blotting was also used to show *in vivo *expression of the proteins, where a section of the harvested muscles at the site of injection was homogenized and then treated with RIPA (50 mM Tris pH 7.4, 150 mM NaCl, 1 mM EDTA, 5 μg/ml Aprotinin, 5 μg/ml Leupeptin, 1% Triton X-100, 1% Sodium deoxycholate, 0.1% SDS) containing 1 mM protease inhibitor PMSF (phenylmethylsulfonyl fluoride). The extracted proteins were mixed with 2× sample buffer (1.25 ml Tris-HCl, pH 6.8, 20% glycerol, 4% SDS, 0.02% (w/v) bromophenol blue, 10% 2-mercaptoethanol) followed by 5-10 min boiling at 95°C. The protein mixtures were subjected to Western blot analysis using primary chicken anti-CAV serum and secondary goat anti-chicken IgY conjugated to alkaline phosphatase.

### Antibody titer to CAV

Serum antibody titers against CAV were determined, pre- and post-vaccination, using IDEXX ELISA kit (IDEXX, Portland, ME, USA). The kit uses an anti-CAV monoclonal antibody in a blocking format ELISA for the detection of antibodies to CAV in chicken serum. Briefly, Samples were diluted at 1:100 dilutions and then added into microtiter wells coated with CAV antigen followed by 60 min incubation at RT. After 3 times washing with washing buffer, anti-CAV monoclonal antibody conjugated with horseradish peroxidase was added and then incubated at RT for 30 min. Following the incubation period, the unreacted anti-CAV conjugate was removed by 3 times washing and TMB substrate solution (100 μl) was then added into the wells followed by 15 min incubation at RT. The reaction was stopped by adding 100 μl of the stop solution and the absorbance value was measured at 650 nm. All sample ODs were normalized to the negative control (S/N = sample OD_650_/negative control OD_650_). The antibody titers were calculated based as the following calculation, log_10 _titer = (S/N-2.72)/-0.64. ELISA titers higher than 1000 were considered as positive.

### Neutralizing antibody titer

To investigate whether the produced antibodies have virus neutralization activity, the collected sera were analyzed for neutralization antibody titer against CAV infection in chicken MSB1 cells. The assay was carried out by the measurement of cell proliferation and viability using WST-1 reagent (Roche, Germany) as described by Moeini *et al. *[12] and Lehtoranta *et al. *[13].

### Cytokines production assay

The serum level of Th1 cytokines, interleukin 2 (IL-2) and interferon γ (IFN-γ) was determined by ELISA kits (Cusabio Biotech, USA). The procedure was following the procedure recommended by the manufacturer. For the reason that no commercial ELISA kits were available for the detection of chicken IL-12, IL-4 and IL-6 which have important roles in the differentiation of CD4^+ ^T cells into Th1 or Th2 cells, transcriptional expression of these cytokines were studied in the spleen of the immunized chickens by RT-PCR using the specific primers listed in Table 1.

### Splenocyte proliferation assay

The proliferation response of the chicken splenocytes to the CAV VP1 protein was studied after immunization. The VP1 protein of CAV was prepared and purified from a recombinant *E. coli *BL21 (DE3) expressing the VP1 and VP2 genes of CAV, synchronously (data not shown). At 10 days of immunization, single cell suspensions were generated from the harvested spleens in PBS-EDTA solution (1X PBS, pH 7.4, 2 mg/ml EDTA) supplemented with 2% penicillin/streptomycin. Red blood cells were removed by washing the cell suspensions with red blood cell lysis solution containing 0.84% NH_4_CL, 0.1% NaHCO_3 _and 1.8 ml of 5% EDTA. The splenocytes were pelleted at 1000 rpm for 10 min and then resuspended in DMEM medium supplemented with 10% fetal bovine serum and 1% penicillin/streptomycin. Viable splenocytes were added to 96-well plates in 0.1 ml at 2 × 10^4 ^cells/well and incubated in triplicate with CAV antigen (purified VP1 protein), mitogen phytohemagglutinin (PHA, Sigma) as positive control, or medium alone as negative control followed by 3 days incubation at 37°C in an atmosphere of 5% CO_2_. The proliferation response was evaluated by BrdU cell proliferation assay kit (Exalpha Biologicals, USA). Data was reported as stimulation indices (SI), which was the mean of experimental wells/mean of antigen free wells (negative control).

### Statistical Analysis

The data was analyzed by *t*-test and statistical significance was set at *P *< 0.05. The results were expressed as means ± standard error of mean. All the analysis was carried out using GraphPad Prism 5 and Windows Microsoft Excel 2007.

## Results

### Construction of plasmids

**Figure 1:**
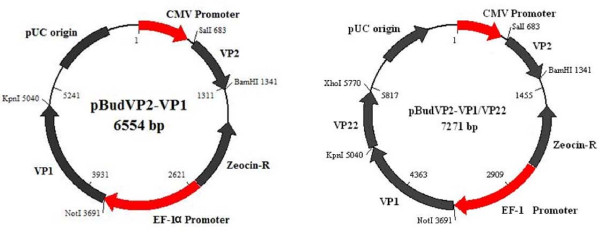
**Map of pBudVP2-VP1 and pBudVP2-VP1/VP22**. pBudVP2-VP1 was constructed by cloning of the CAV VP1 gene into the *Not*I and *Kpn*I of EF-1α MCS and VP2 gene into the *Sal*I and *Bam*HI sites of CMV MCS of pBudCE4.1. To generate pBudVP2-VP1/VP22, the MDV-1 VP22 DNA fragment was inserted into the *Kpn*I and *Xho*I cloning sites of the constructed pBudVP2-VP1 plasmid in the frame with the CAV VP1 gene.

The VP1 and VP2 genes of CAV isolate SMSC-1 were amplified and cloned into the vector pBudCE4.1 to construct pBudVP2-VP1 encoding VP1 and VP2, synchronously. The pBudVP2-VP1/VP22 DNA plasmid was also constructed by insertion of the VP22 gene of MDV-1 into pBudVP2-VP1 in the frame with VP1. The plasmid constructs had the correct orientation and the correct order of the reading frames (data not shown). The final constructs are shown in Figure 1.

### In vitro characterization of the constructs

**Figure 2:**
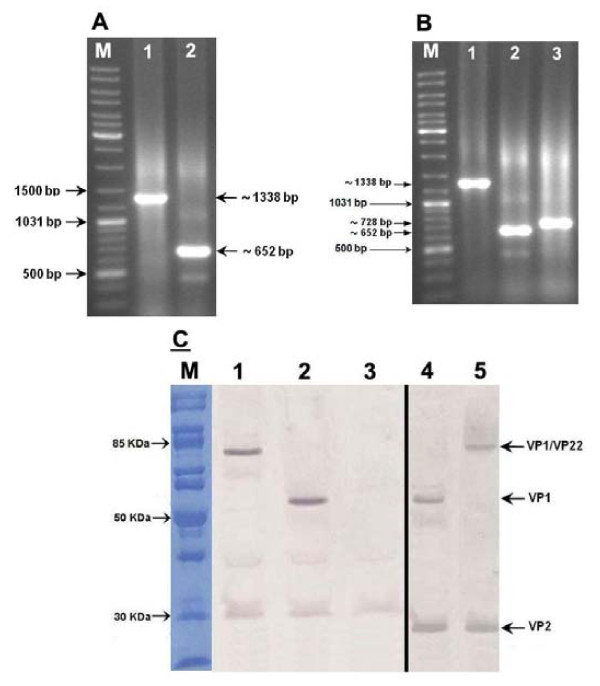
***In vitro *expression analysis of the constructs in chicken MSB1 cells by RT-PCR (A and B) and Western blotting (C)**. (A) RT-PCR results for VP1 and VP2 (lanes 1 and 2, respectively) in the pBudVP2-VP1-transfected cells. (B) RT-PCR results for VP1, VP2 and VP22 (lanes 1-3) in the pBudVP2-VP1/VP22-transftected cells; M: GeneRuler™DNA Ladder Mix (Fermentas, Canada). (C) Western blotting results by using chicken anti-CAV (lanes 1-3) or anti-His (lanes 4 and 5) in the cells transfected with pBudVP2-VP1/VP22 (lane 1 and 5), pBudVP2-VP1 (lane 2 and 4) or in non-transfected cells (lane 3).

*In vitro *expression of the DNA plasmids was carried out in chicken MSB1 cells. RT-PCR analysis of the DNA-free RNAs using the respective primers for VP1, VP2 and VP22 revealed the presence of the mRNA of the genes in the transfected cells indicating the successful *in vitro *transcriptional expression of the constructs (Figure 2A &2B).

*In vitro *translation of the DNA vaccines was confirmed by Western blot analysis of the transfected MSB1 cells (Figure 2C). The expression of VP1 (~56 KDa) and VP1/VP22 (~82 KDa) proteins were detected in the cells transfected with pBudVP2-VP1 or pBudVP2-VP1/VP22, respectively using both anti-His and anti-CAV primary antibodies. The VP2 protein (~27 KDa) was detected in the both transfected cells using anti-His monoclonal antibody (Figure 2C, lanes 4 and 5).

**Figure 3:**
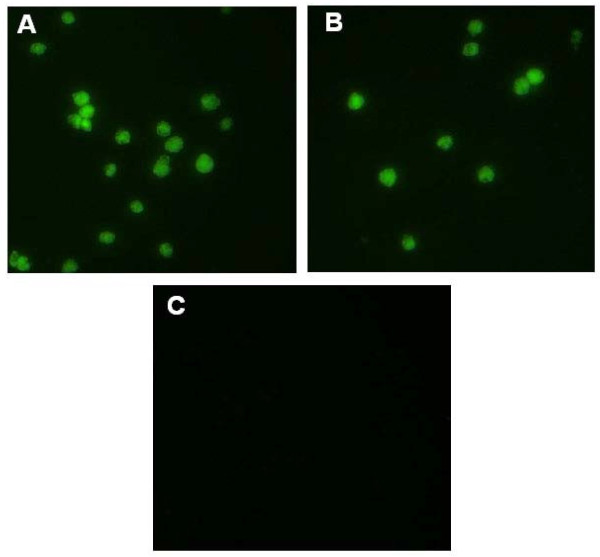
**Indirect immunofluorescence analysis**. MSB1 cells were transfected with the recombinant plasmids by using Lipofectamine 2000 reagent and 2 days after transfection, transient expression of the encoded proteins was explored by indirect immunofluorescence using primary anti-CAV polyclonal antibody. (A) Cells transfected with pBudVP2-VP1; (B) Cells transfected with pBudVP2-VP1/VP22; and (C) Cells transfected with the parental plasmid.

Indirect immunofluorescence using chicken anti-CAV serum followed by FITC-conjugated anti-chicken IgY also revealed the expression of the encoded proteins. Cells transfected with pBudVP2-VP1 or pBudVP2-VP1/VP22 exhibited bright cytoplasmic fluorescence (Figure 3A &3B) compared with the cells transfected with the parental plasmid that showed no fluorescence (Figure 3C) indicating that the VP1 protein linked to the MDV-1 VP22 gene capable to recognize and interact with anti-CAV antibody.

### In vivo evaluation of the DNA vaccines

**Figure 4:**
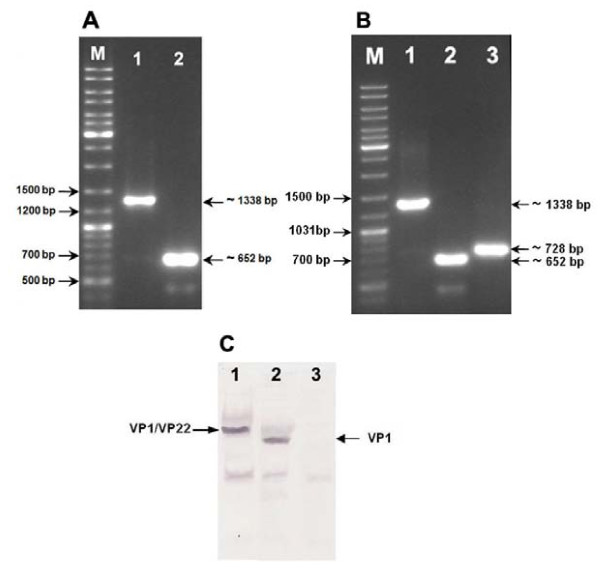
***In vivo *expression analysis of the viral genes in the injected chickens by RT-PCR (A & B) and Western blotting (C)**. (A) RT-PCR results for the chicken injected with pBudVP2-VP1. (B) RT-PCR results for the chicken injected with pBudVP2-VP1/VP22; M: GeneRuler™DNA Ladder Mix (Fermentas, Canada). (C) Western blotting results in the chicken injected with pBudVP2-VP1/VP22 (lane 1), pBudVP2-VP1 (lane 2) or parental plasmid (lane 3).

The DNA plasmids were tested in SPF chickens via intramuscularly injection. Chicken were boosted twice, at 2 weeks intervals. Expression analysis of the genes by RT-PCR revealed the transcriptional expression of VP1, VP2 and MDV-1 VP22 in the skeletal muscle at the site of injection (Figure 4A &4B). Furthermore, Western blot analysis showed the expression of VP1 and VP1/VP22 proteins at the site of injection in the chickens vaccinated with pBudVP2-VP1 or pBudVP2-VP1/VP22, respectively (Figure 4C).

### Serum antibody titer and VN titer to CAV in the vaccinated chickens

**Figure 5:**
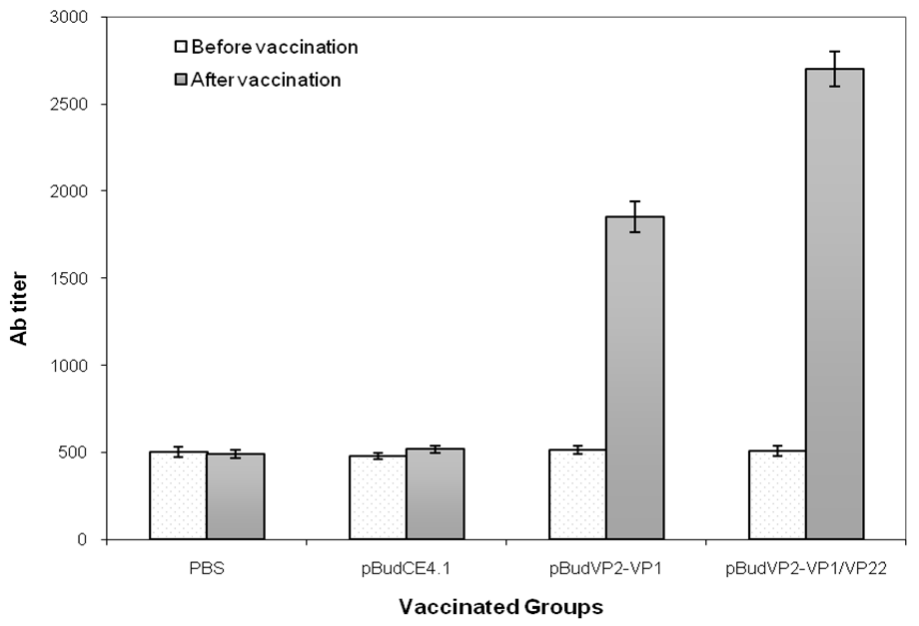
**Antibody responses to CAV as determined by ELISA pre- and post-vaccination**. Control groups were inoculated with PBS and pBudCE4.1 plasmid. Antibody titers higher than 1000 were considered positive. The error bars indicate the standard errors of the means.

Serum antibody titer to CAV was determined in the chickens pre- and post-vaccination by ELISA. As shown in Figure 5 vaccinated Chickens with pBudVP2-VP1/VP22 showed a significant increase (P < 0.05) in antibody titer (2700 ± 100) compared with the pBudVP2-VP1-vaccinated chickens (1853 ± 89). No detectable response was detected in the control groups injected with PBS or the parental plasmid.

To investigate the neutralization activity of the serum antibodies, virus neutralization test was carried out in MSB1 cells. According to the results summarized in Table 2 both groups showed positive virus neutralization activity with titer ranging from 1:256 to 1:512. From eight pBudVP2-VP1/VP22-injected samples, 6 samples had VN titer of 1:512, while only one of the seven pBudVP2-VP1-injected samples showed VN titer of 1:512.

**Table 2:** Neutralization activities of the serum antibodies in the vaccinated groups.

		Virus neutralization test		
		
Sera groups	No. of samples	No. of positive	VN antibody titer	VN status
pBudVP2-VP1/VP22	8	2	1:256	positive
pBudVP2-VP1	7	6	1:512	positive
parental plasmid	2	6	1:256	positive
PBS	2	1	1:512	positive
		-	0	Negative
		-	0	Negative

### Cell-mediated responses after DNA vaccination

**Figure 6:**
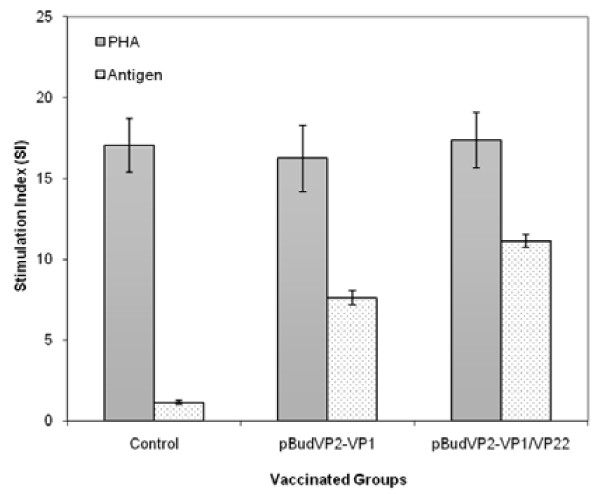
**Proliferative induction of splenocytes from the immunized chickens**. Ten days after the last injection, proliferation response was evaluated by BrdU cell proliferation assay kit as described in material and methods. Cell induced with mitogen phytohemagglutinin (PHA) were used as positive control. Data was reported as stimulation indices (SI). SI greater than two was considered positive.

Cell-mediated immunity was evaluated in the vaccinated group through cytokines evaluation and *in vitro *proliferation assay of splenocytes pre- and post-immunization. Splenocytes of the immunized chickens were stimulated with the antigenic VP1 protein, or PHA as positive control. The assay showed positive proliferation response in the both vaccinated groups, although the VP1-induction level in the pBudVP2-VP1/VP22-immunized group was significantly higher (mean SI 11.12) compared to the pBudVP2-VP1 group (mean SI 7.63) (Figure 6). The controls inoculated with parental plasmid showed negative responses (SI < 2) to the CAV VP1 protein.

**Figure 7:**
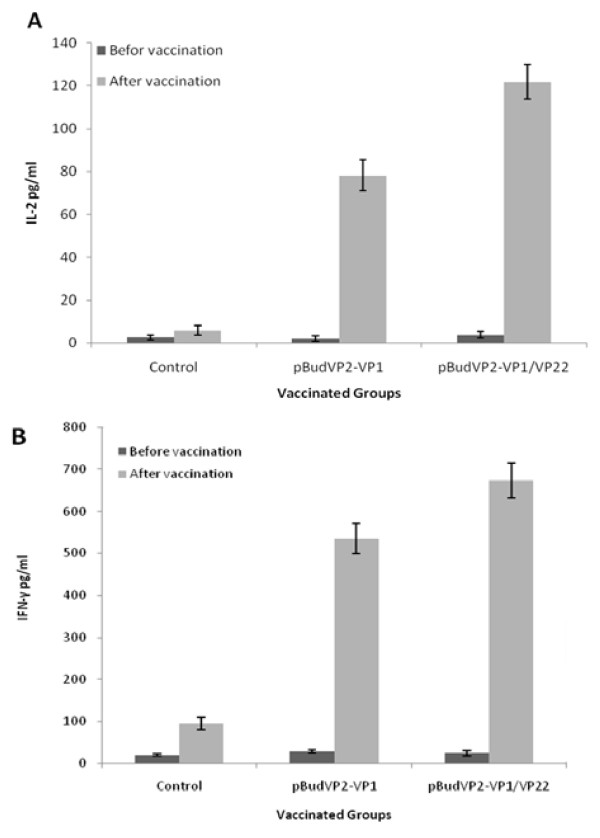
**Serum level of IL-2 and IFN-γ in the vaccinated chickens**. The serum levels of Th1 cytokines, IL-2 (A) and IFN-γ (B) were determined in pBudVP2VP1-, pBudVP2-VP1/VP22- and the control group. The results showed higher levels of IL-2 and IFN-γ in the immunized groups compared to those from the control group.

To explore whether the DNA vaccines lead to the induction of Th1 or Th2 response, serum levels of Th1 cytokines, IL-2 and IFN-γ were evaluated by ELISA kits. No significant differences were observed between the pBudVP2-VP1- and pBudVP2-VP1/VP22-vaccinated groups and controls pre-immunization, while the serum level of IL-2 and IFN-γ was significantly (P < 0.05) higher in the vaccinated groups post-vaccination when compared to the control group (Figure 7). However, compared with the pBudVP2-VP1 group, the pBudVP2-VP1/VP22 group showed higher levels of IL-2 and IFN-γ (P < 0.05). Furthermore, transcriptional expression analysis showed the presence of IL-12 mRNA in the vaccinated groups, whereas the mRNA of IL-4 and IL-6 was not detected (results not shown).

## Discussion

According to the previous studies [6,14,15] and the finding of our group [12], co-synthesis of the VP1 and VP2 proteins is required to produce the essential neutralizing form of VP1 resulting in the efficient induction of antibody response to CAV, whereas separate expression of VP1 does not. In fact, VP2 may play a role as a scaffold protein during virion assembly, but is removed in the next step [14,15]. Therefore, pBudVP2-VP1 plasmid co-expressing the VP1 and VP2 genes of CAV was developed as a DNA vaccine against CAV. To explore whether the linkage of MDV-1 VP22, as a tegument protein with a property of intercellular transport, to CAV VP1 protein lead to enhance the potency of the DNA vaccine, the pBudVP2-VP1/VP22 plasmid encoding the fusion protein VP1/VP22 and VP2 protein was also constructed.

*In vitro *analysis of the constructs revealed the successful transcriptional and translational expression of the genes in MSB1 cells. In agreement with the immunoblotting results, immunofluorescence staining of the transfected cells showed the reaction of the VP1 and the fusion protein, VP1/VP22 with anti-CAV polyclonal antibody resulting in bright cytoplasmic fluorescence. These observations indicated that the VP1 protein has proper secondary folding to induce immune system to CAV. The plasmid constructs were then tested in SPF chickens. *In vivo *expression analysis matched up well with *in vitro *findings, confirmed the transcriptional and translation expression of the genes in the immunized chickens.

To investigate the ability of the linkage MDV-1 VP22 to CAV VP1 to enhance the potency of the DNA vaccine, immune responses were evaluated in the chickens vaccinated with the plasmid encoding VP1/VP22 and the results were compared with those from the chickens vaccinated with pBudVP2-VP1. Antibody titer to CAV was significantly higher (P < 0.05) in the group vaccinated with pBudVP2-VP1/VP22 in comparison with the group vaccinated with pBudVP1-VP2. Virus neutralization (VN) test indicated positive neutralization activity of the antibodies in the both vaccinated groups with moderate protective titer ranging from 1:256 to 1:512. However, the number of samples with VN titer of 1:512 was obviously higher in the pBudVP2-VP1/VP22-vaccinated group. Similarly such presence of neutralizing antibodies against CAV has been shown to provide protection against CAV in young chickens [16-21]. Nevertheless, in order to confirm this issue, it is suggested that breeder flocks producing day-old chicks with maternal antibodies be vaccinated for virus challenge.

Cytokine assay by ELISA showed high level of IL-2 and IFN-γ in the both vaccinated groups post-immunization, although their levels were higher in the pBudVP2-VP1/VP22 group. Furthermore, the expression of IL-12 which triggers the differentiation of Th1 [22] was detected by RT-PCR in the spleen of the both groups, whereas IL-4 and IL-6 which are involved in differentiation of Th2 [22,23] were not detected in any samples. These observations indicate a pattern of cytokine production that most closely promotes the Th1 T-helper cell responses which are known to be involved in cellular-mediated immunity [22,24].

To further investigation whether the linkage of MDV-1 VP22 lead to enhance antigen-specific immune responses, we compared antigen-stimulated proliferative response in the splenocytes of the vaccinated groups. Compared to the pBudVP2-VP1-vaccinated group, the pBudVP2-VP1/VP22-vaccinated group exhibited a significant increase in the proliferation induction of splenocytes indicating higher VP1-specific immune responses in the presence of the MDV-1 VP22 protein.

## Conclusions

The results of the present study showed that the fusion of the MDV-1 VP22 to the target gene, CAV VP1, could significantly increase CAV-specific immune responses. This view is supported by Hung *et al. *[7], who showed the capacity of the VP22 protein of Marek's disease virus type-1 (MDV-1) to improve DNA vaccine potency by facilitating intercellular spreading of the linked protein.

## Competing interests

HM is a graduate student of Universiti Putra Malaysia (UPM). ARO, RAR and KY are employees of the same institution. The university holds the rights for all the financial benefits that may result from this study. The authors have no any competing interests with this study.

## Authors' contributions

HM designed the study, carried out the experiments and drafted the manuscript. ARO and RAR participated in the design of the study. KY conceived the study, participated in its design and co-ordination. All authors read and approved the final manuscript.
